# Electroacupuncture Regulates Sympathetic Nerve Through the NTS^Glu^ ‐RVLM Circuit to Relieve Spontaneous Pain in SNI Rats

**DOI:** 10.1111/cns.70327

**Published:** 2025-03-27

**Authors:** Wen Chen, Xin Ma, Yi‐Ming Fu, Cun‐Zhi Liu, Hong‐Ping Li, Guang‐Xia Shi

**Affiliations:** ^1^ International Acupuncture and Moxibustion Innovation Institute Beijing University of Chinese Medicine Beijing China; ^2^ School of Acupuncture‐Moxibustion and Tuina Shandong University of Traditional Chinese Medicine Jinan China

**Keywords:** electroacupuncture, mechanical pain, neuropathic pain, nucleus tractus solitarii, rostral ventrolateral medulla, spontaneous pain, sympathetic nerve

## Abstract

**Aim:**

Patients suffering from neuropathic pain often experience sympathetic dysfunction. Acupuncture has shown promise in alleviating pain and modulating the activity of the autonomic nervous system. This study aims to explore the potential mechanism through which electroacupuncture (EA) modulates sympathetic nerves to alleviate neuropathic pain.

**Methods:**

Spared nerve injury (SNI) was utilized to induce neuropathic pain. EA was administered at acupoints Huantiao and Yanglingquan for 30 min every other day after SNI. Pain behavior was evaluated using paw withdrawal thresholds (PWTs) and spontaneous pain scores. Various techniques including immunofluorescence, viral tracing, electrophysiology, and chemogenetic manipulations were employed to investigate the impact of EA on the sympathetic nerves and pain behaviors, specifically through the nucleus tractus solitarii (NTS)^Glu^‐rostral ventrolateral medulla (RVLM) circuit.

**Results:**

In SNI rats, EA alleviated both mechanical and spontaneous pain, diminished sympathetic nerve excitability, and inhibited sympathetic nerve sprouting within the dorsal root ganglia (DRG), reduced the excitability of glutamatergic neurons in the NTS which project to the RVLM. Chemogenetic inhibition of the NTS^Glu^‐RVLM circuit produced the same effect as EA in spontaneous pain, sympathetic nerve excitability, extracellular discharge frequency in RVLM, but not in mechanical pain. Similarly, chemogenetic activation of the NTS^Glu^‐RVLM circuit negated the analgesic effects of EA on spontaneous pain while not affecting mechanical pain.

**Conclusions:**

This study suggested that EA alleviates spontaneous pain rather than mechanical pain by regulating the sympathetic nerve activity via the NTS^Glu^‐RVLM circuit.

## Graphical abstract

Effect of EA on the NTS^Glu^‐RVLM circuit in the regulation of sympathetic nerve activity in SNI rats.

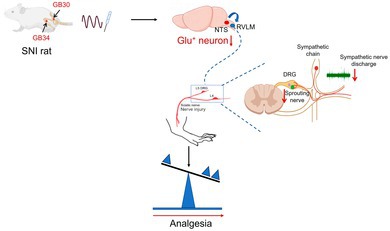

## 1 Introduction

Neuropathic pain is a chronic pain caused by the injury or disease of the somatosensory nervous system and is characterized by allodynia, hyperalgesia, and spontaneous pain [1]. Epidemiological studies have shown that the prevalence in the general population may be as high as 7%–8%, accounting for 20%–25% of individuals with chronic pain [2, 3]. Since the last century, studies have shown that the sympathetic nervous system was involved in neuropathic pain [4, 5]. Sympathetic nerve activity was increased in neuropathic pain patients, and sympathetic blockade relieved the pain [6, 7]. After peripheral nerve injury, postganglionic sympathetic nerve sprouting occurs and forms a “basket‐like” structure around the sensory neurons in the dorsal root ganglia (DRG), resulting in sympathetic‐sensory coupling. This structure amplifies noxious information on the body surface and promotes pain [8, 9, 10]. When the sympathetic nerves were removed, the sprouting fibers almost entirely disappeared, and pain was also significantly alleviated [11]. But sympathetic nerves play different roles in distinct types of pain sensations [12]. In the spinal nerve ligation (SNL) model, sympathectomy was effective only when the animals showed severe mechanical hypersensitivity but had no effect on a lesser degree of mechanical hypersensitivity [13]. In the spared nerve injury (SNI) model, surgical lumbar sympathectomy had no effect on mechanical allodynia and mechanical hyperalgesia but significantly attenuated cold allodynia [14]. The mechanism underlying the phenomenon still has not been completely clear.

Many brain regions contributed to the regulation of the autonomic nervous system, including the nucleus tractus solitarii (NTS) [15]. NTS is a sensory nucleus located in the dorsomedial region of the medulla oblongata [16] and is considered an integral part of the endogenous pain‐modulatory system [17]. The previous studies had shown that NTS was required for the maintenance of sympathetic facilitation in some diseases [18, 19]. In chronic pain conditions, the glutamatergic NTS neurons modulated depression‐like behaviors but not hypersensitive behaviors that were related to pain [17]. The role of glutamatergic NTS neurons on sympathetic activity in chronic pain has not been fully understood. Neurons in NTS integrate sensory signals from the viscera and body, then relay the information to other brain regions, including neurons in the rostral ventrolateral medulla (RVLM) [20, 21]. RVLM contains presympathetic neurons and controls sympathetic outflow [21]. Notably, tonically active glutamatergic inputs to the RVLM were enhanced in neuropathic pain, which contributes to high sympathetic activity [22]. Furthermore, the glutamatergic neurons in NTS can excite the RVLM and subsequently increase sympathetic nerve excitability [23]. But the role of the NTS^Glu^‐RVLM circuit on sympathetic activity in neuropathic pain remains to be elucidated.

Acupuncture, a crucial element of traditional Chinese medicine, is widely utilized as a nondrug therapy on a global scale. Existing research shows that acupuncture has an effect on the regulation of autonomic nerves [24, 25]. In colorectal distension (CRD) rats, electroacupuncture (EA) could regulate CRD‐related neurons in the NTS, which mediates EA analgesia on visceral pain [26]. More than that, Studies have confirmed that EA activated NTS neurons that directly project to the RVLM, suggesting a potential interaction between NTS and RVLM during EA [27]. The projection from NTS to RVLM has an effect on the activity of the RVLM and regulates the balance of the autonomic nervous system [28]. However, it is unclear whether EA can regulate RVLM through glutamatergic neurons in the NTS under neuropathic pain conditions, thereby inhibiting sympathetic nerves and exerting an analgesic effect.

In this study, we found that in the SNI rat model, EA decreased the excitability of NTS glutamatergic neurons projecting to the RVLM, reduced sympathetic nerve excitability, and diminished sympathetic sprouting in the DRG. We also validated that the NTS^Glu^‐RVLM circuit in SNI rats contributed to the phenomenon by using chemogenetic methods. Besides, there was an interesting finding that the intervention of the circuit only had an effect on spontaneous pain while not on mechanical pain.

## 2 Materials and Methods

### 2.1 Animals

Healthy male Sprague Dawley rats weighing 180–220 g were purchased from Beijing Vital River Laboratory Animal Technology Co. Ltd. All experiments adhered to the guidelines of the Committee for Research and Ethical Issues of the International Association for the Study of Pain (IASP) [29]. This research was approved by the Ethics Committee of Beijing University of Chinese Medicine, Ethics Approval No: BUCM‐2024011502‐1031. Rats were raised in a controlled environment (temperature 18°C–22°C, humidity 40%–70%) with a 12 h light–dark cycle, with water and food available ad libitum. The animals were habituated to the environment for 7 days before all experiments.

### 2.2 Establishment of spared nerve injury (SNI) model

The SNI model was created using a procedure similar to that described by Decosterd and Woolf [30]. Rats were anesthetized with 2% isoflurane in an oxygen/air mixture, and the left sciatic nerve was exposed. The common peroneal and tibial nerves were tightly ligated 8 mm distal to the sciatic trifurcation, and the nerve was transected distal to the ligation. To prevent regeneration, 2 mm of the distal stump of the cut nerve was removed. Careful attention was given to preserving the integrity of the sural nerve. Sham surgery rats underwent the same surgical procedure as the SNI rats, but the nerves were left intact. To maintain consistent experimental conditions, all surgical operations were performed by the same individual. Following surgery, the rats were placed in their familiar standard environment, where they had previously acclimated, and were provided with unrestricted access to food and water.

### 2.3 EA Intervention

During the EA stimulation, the rat was restrained but left in an awake state. EA was performed on the first day after SNI. Two stainless steel acupuncture needles (0.25 × 25 mm) were inserted at a depth of 8–10 mm into the left Huantiao (GB30) acupoint and 3–5 mm into the left Yanglingquan (GB34) acupoint. The electrical stimuli were administered using a HANS‐200A Acupuncture Point Nerve Stimulator (Nanjing, China), which, with a frequency of 2 Hz and the EA stimulation intensity was increased in gradient by 1‐2‐3 mA, each stimulation intensity lasting for 10 min. The rats treated once every other day for 42 days. The acupoints in the SNI + sham EA group were the same as those in the SNI + EA group. Only superficial acupuncture (at a depth of 0.5–1 mm) was performed without EA stimulation. Rats in the Sham group and SNI group underwent grasping and fixation similar to those in the SNI + EA group but without EA stimulation.

### 2.4 Withdrawal Threshold to Mechanical Stimulation

The 50% mechanical paw withdrawal threshold (PWT) was determined using the “up and down” method [31]. In brief, animals were placed on a metal mesh floor covered with an inverted clear plastic cage (18 × 8 × 8 cm) and the plantar surface of the paw was stimulated with a series of ascending force von Frey monofilaments (Stoelting, Wood Dale, IL). Eight von Frey monofilaments with approximately equal logarithmic incremental (0.224) bending forces were chosen (0.40, 0.60, 1.00, 2.00, 4.00, 6.00, 8.00, and 15.00 g). Each trial started with a von Frey force of 2.00 g delivered perpendicularly to the lateral plantar surface of the left hindpaw. An abrupt withdrawal of the foot during stimulation or immediately after the removal of the filament was recorded as a positive response. Whenever there was a positive or negative response, the next weaker or stronger filament was applied, respectively. This procedure was done until six stimuli after the first change in response had been observed. The 50% PWT was calculated using the following formula: 50% PWT (g) =  (10^X^_f_+^κδ^)/10^4^ where *X*_*f*_ is the value of the final von Frey filament used (in log units), *κ* is a value measured from the pattern of positive/negative responses, and *δ* = 0.224, which is the average interval (in log units) between the von Frey filaments. The 50% PWT of an allodynic rat is defined as 4.0 g, that is, non‐noxious tactile stimulus induce the withdrawal response [32].

### 2.5 Spontaneous Pain Behavior

To assess spontaneous pain behavior, animals were placed in plexiglass containers and recorded for 60 min. The recorded video was then played back in slow motion to accurately count the number of instances of licking and flinching/shaking/lifting with the ipsilateral hind paw. Movements related to exploration, locomotion, body repositioning, and grooming were not considered. Each instance of licking was assigned two points, while each instance of flinching/shaking/lifting was assigned one point. The total points accumulated during the 1‐h recording were used as the spontaneous pain score [33, 34].

### 2.6 Stereotaxic Injection

Cre‐dependent adeno‐associated viruses (AAVs) were used to manipulate neural activity in the NTS^Glu^‐RVLM circuit in this study. AAV injections and cranial window implantations were performed as previously described [35, 36]. The rats were anesthetized with 4% isoflurane, maintained at 2% isoflurane, and positioned in a stereotaxic apparatus (RWD, Shenzhen, China). The skull was exposed via a small incision, and two small holes were drilled (0.50 mm drill bit) into the skull to introduce a microinjection glass pipette into the NTS (AP, −12.75 mm; ML, −1 mm; DV, −6.5 mm) in a volume of 200 nL for each side at 1 nL/s and into the RVLM (AP, −12.25 mm; ML, −2 mm; DV, −7.8 mm) in a volume of 200 nL at 1 nL/s. The syringe was slowly retracted after an additional 10 min diffusion of the virus. The skin was sutured, and the animals were allowed to recover in prewarmed cages before returning to the home cage. For NTS^Glu^‐RVM circuit tracing, a viral approach was performed to label the NTS glutamatergic neurons projecting to the RVLM: we injected rAAV‐CaMKIIα‐mCherry into the NTS.

**FIGURE 1:**
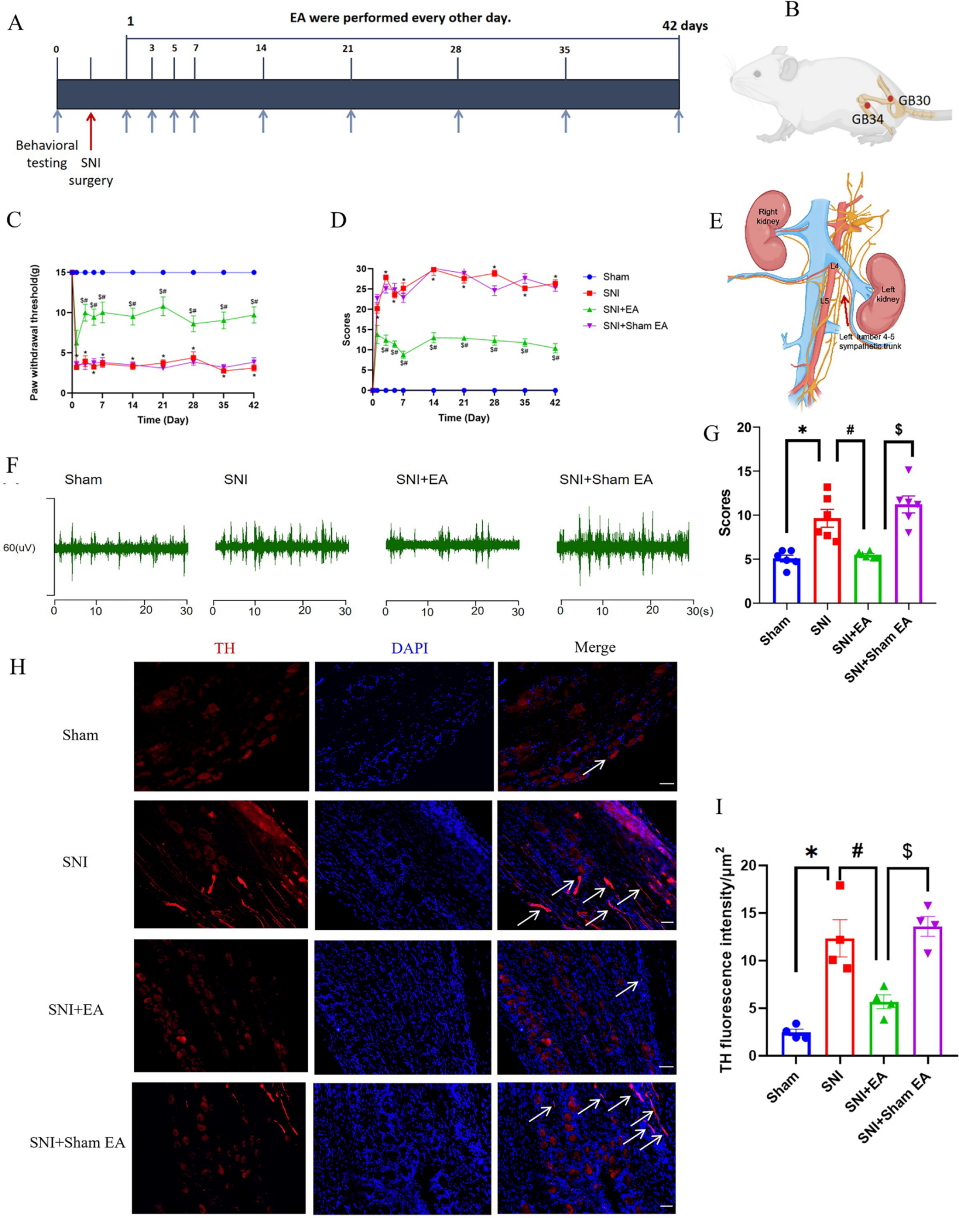
EA attenuates SNI‐induced pain behavior and sympathetic nerve activity. (A) Timeline of the SNI surgery, EA, and behavioral testing. (B) Schematics showing the left hindlimb GB30 and GB34 acupoints. (C) Time course of EA on the mechanical pain threshold of SNI rats (*n* = 10/group), using the Kruskal‐Wallis test. (D) Time course of EA on spontaneous pain scores of SNI rats (*n* = 10/group), using the Kruskal‐Wallis test. (E) Lumbar sympathetic trunk schematic diagram. (F) Representative images of sympathetic nerve discharge from the sham, SNI, SNI + EA, and SNI + sham EA groups. (G) The sympathetic nerve excitability was significantly decreased after EA (*n* = 6/group), one‐way ANOVA with Dunnett's T3 test. (H) Representative images of sympathetic sprouting in DRG from sham, SNI, SNI + EA, and SNI + sham EA groups; scale bar, 50 μm, the arrows indicated TH^+^ nerve fibers. (I) The sprouting was significantly reduced after EA (*n* = 4/group), one‐way ANOVA with Tukey test. *p < 0.05 compared with the sham group, #*p* < 0.05 compared with the SNI group, $*p* < 0.05 compared with SNI + sham EA group.

In order to detect the role of the circuit in EA analgesia, we used chemogenetic manipulation. Like other reports, it indicated that ipsilateral EA stimulation could alleviate knee osteoarthritis (KOA) pain behavior by regulation of ipsilateral sympathetic [37], in Figure 1, the results also showed that ipsilateral EA stimulation could alleviate pain behavior and ipsilateral sympathetic activity. Besides, as an important area for regulation of sympathetic outflow, there were reports that indicated that RVLM had predominantly ipsilateral innervation of sympathetic nerves [38, 39], so we injected rAAV‐DIO‐hM3Dq/hM4Di‐mCherry or rAAV‐DIO‐mcherry into the left NTS and Retro‐rAAV‐CaMKIIα‐Cre virus into the left RVLM, to specifically label the NTS^Glu^‐RVLM neural circuit, and intraperitoneal injection of CNO (1 mg/kg) half an hour before EA stimulation to activate or inhibit the neural activities. All viruses were purchased from Brain Case (Wuhan, China).

### 2.7 Extracellular Single‐Unit Recordings

Rats were deeply anesthetized with 2% isoflurane in an oxygen/air mixture and placed in a stereotaxic apparatus (RWD, Shenzhen, China). According to the above method, a craniotomy was performed to expose the brain above the hypothalamic NTS and RVLM. Then, an aseptic surgical procedure was performed to implant a single electrode (resistance: 1 M) (KD‐SC, KedouBC), with a 100 μm inner electrode diameter and a 50 mm interelectrode distance, into a target cortical area. Slowly advance the electrode within the target brain area to look for neurons with stable discharge. The frequency of the neuronal discharge was analyzed with special software (SpikeHistogram, AD Instruments). Impair the electrode (1 mA, 10 s) to mark the site of the last recorded neuron [40].

### 2.8 Immunohistochemistry

Forty‐two days after the SNI operation, rats were anesthetized with 2% isoflurane and subjected to intracardiac perfusion with 0.9% saline followed by 4% paraformaldehyde (PFA). The L4–L6 DRGs and the brain were postfixed in 4% PFA for 6–8 h. The tissues were subsequently removed and dehydrated in 20% and 30% sucrose successively until they completely sunk to the bottom of the container. Sections were cut at 20 μm for DRGs and brain on a cryostat (CM1950, Leica). The sections were blocked in 5% normal donkey serum and 0.2% Tween 20 in phosphate buffer (PB) for 1 h at 37°C After blocking, the DRG sections were incubated overnight with the primary antibody, which includes mouse antineurfilament‐200 (anti‐NF200) (1:400, 2836S, Cell Signaling Technology), rabbit anticalcitonin gene‐related peptide (CGRP) (1:400, #14959, Cell Signaling Technology), FITC‐conjugated isolectin B4 (IB4) (1:400, L2140, Sigma‐Aldrich) to label sensory neurons in the DRGs, and sheep antityrosine hydroxylase (anti‐TH) (1:1000, PA1‐4679, Invitrogen) to visualize sympathetic fibers in the DRGs. The brain sections were incubated overnight with the primary antibody rabbit anti‐c‐Fos (1:200, 2250S, Cell Signaling Technology) to label activated neurons in the NTS and RVLM, mouse anticalcium‐CaM‐dependent protein kinase II (CaMK II) (1:200, 66843‐1‐Ig, Proteintech), and mouse anti‐tyrosine hydroxylase (TH) (1:100, 66843‐1‐Ig, Proteintech) to label glutamatergic neurons in the NTS and presympathetic neurons in the RVLM, respectively. After overnight incubation with primary antibodies at 4°C, the slides were washed three times in PB and incubated with appropriate Alexa Fluor‐conjugated secondary antibodies (1:1000, Jackson, USA) for 1 h at room temperature. Then sections were counterstained with the nuclear marker DAPI (100 ng/mL, Sigma‐Aldrich) and captured with a confocal imaging system (FV1200, Olympus, Tokyo, Japan).

### 2.9 Sympathetic Nerve Activity Recording

To assess sympathetic nerve activity in SNI rats, L4–L5 sympathetic nerve trunks were dissociated and recorded using a platinum wire electrode (50 μm). Rats were anesthetized with 10% urethane (1.2 mg/kg, ip) and fixed on the operating table in a supine position. An incision was made along the midline of the abdomen to expose the abdominal aorta. Then the operation was switched to a microscope, and a glass minute needle was used to trace the abdominal aorta longitudinally. The retroperitoneum was separated and mobilized to both sides to expose the left lumbar sympathetic nerve. A plastic wrap was used to isolate the surrounding tissue under the nerve, and paraffin oil was dropped to keep the nerve moist. Fiber tweezers were used to peel off the mucosa and epineurium about 2 mm long on the nerve surface. The electrode was inserted into the sympathetic nerve at about 30°. The signal was amplified by a preamplifier (AM‐1800; A–M Systems, Sequim, WA, USA) and collected through the Powerlab data acquisition system using LabChart 8.0 software (ADInstruments, NSW, Australia). Spike frequency was analyzed using spike histograms of LabChart 8.0 (gain, 1000 Hz; low‐cut filter, 100 Hz; high‐cut filter, 1000 Hz) [41].

### 2.10 Statistical Analysis

Statistical analyses were performed with IBM SPSS Statistics 21.0 and GraphPad Prism 8.0 for Windows (GraphPad Software). Shapiro–Wilk tests were used to assess normality in the distribution (Gaussian distribution) for each group. For normally distributed data, One‐way analysis of variance (ANOVA) with the Tukey method (equal variance) or Tukey–Kramer method (equal variance, unequal number of cases between groups) or Dunnett's T3 method (unequal variance) was used. Additionally, Kruskal–Wallis was employed to compare non‐normally distributed data. All data are reported as mean ± SEM, and differences with *p* < 0.05 were considered statistically significant.

## 3 Results

### 3.1 EA Improved Pain Behaviors and Inhibited Sympathetic Nerve Activity in SNI Rats

To investigate the impact of EA stimulation on pain behaviors in rats suffering from SNI, rats in the SNI + EA group received 2 Hz EA treatment every other day for 42 days post‐SNI surgery (Figure 1A). The positioning of acupoints is illustrated in Figure 1B. PWT and spontaneous pain scores were assessed. Results showed a significant decrease in PWT (*p* < 0.05; Figure 1C) and a notable increase in spontaneous pain scores (*p* < 0.05; Figure 1D) on the first day post‐SNI, which persisted throughout the modeling period, indicating the successful establishment of SNI‐induced neuropathic pain. EA treatment effectively raised PWT and decreased spontaneous pain scores (*p* < 0.05) from day 3 to 42 after surgery (Figure 1C,D), demonstrating that 2 Hz EA stimulation can alleviate mechanical and spontaneous pain induced by SNI. At the same time, using electrophysiological recording techniques to analyze the L4–L5 sympathetic nerves excitability (Figure 1E), we found that the sympathetic nerve excitability was enhanced in SNI rats, and EA treatment could reverse the increase induced by the SNI operation (Figure 1F,G). Sympathetic sprouting also occurred during neuropathic pain conditions in many researches [42, 43]. In our studies, we found the same results as previously described. In SNI rats, obvious sympathetic‐sensory coupling was observed up to 6 weeks after the SNI operation (Figure S1A), 42 days after the SNI operation, the expression of TH was notably increased, and EA treatment significantly decreased the expression of TH (Figure 1H,I). Also, as described in other reports TH^+^ nerve fibers predominantly encircled NF200^+^ neurons, rather than CGRP^+^ and IB4^+^ neurons (Figure S1B). The results suggested that the sympathetic nerve might be involved in EA analgesia.

### 3.2 EA Inhibited NTS and RVLM Neuronal Activities

**FIGURE 2:**
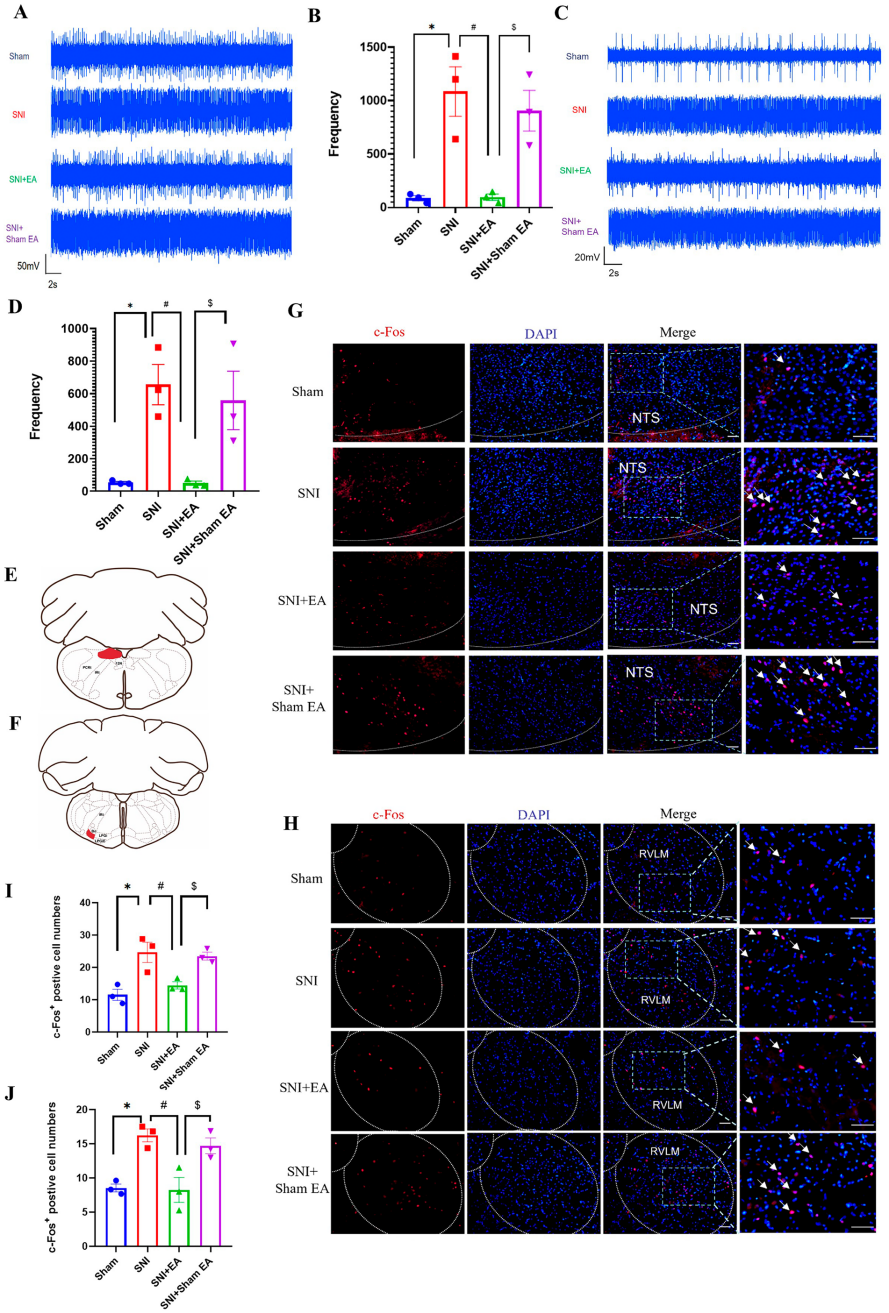
EA inhibited the excitability of NTS and RVLM. Representative images of extracellular electrophysiology of NTS (A) and RVLM (C) from the sham, SNI, SNI + EA, and SNI + sham EA groups. The discharge frequency of NTS (B) and RVLM (D) was significantly decreased after EA (*n* = 3/group), one‐way ANOVA with Tukey in (B), Kruskal–Wallis test in (D). The location of NTS (E) and RVLM (F) for immunofluorescence imaging. Representative images of c‐Fos (red) in the NTS (G) and RVLM (H) from the sham, SNI, SNI + EA, and SNI + sham EA group; scale bar, 50 μm, the arrows indicated c‐Fos^+^ neurons. The number of c‐Fos‐positive neurons in the NTS (I) and RVLM (J) was significantly decreased after EA (*n* = 3/group), one‐way ANOVA with Tukey test in I and J. *p < 0.05 compared with the sham group, #*p* < 0.05 compared with the SNI group, $*p* < 0.05 compared with the SNI + sham EA group.

Previous researches reported that NTS and RVLM regulated the sympathetic nerve activity [22]. In order to assess the role of NTS and RVLM on sympathetic in EA analgesia, extracellular discharges in NTS and RVLM were analyzed. Results indicated a notable increase in discharge frequency in the NTS (*p* < 0.05; Figure 2A,B) and RVLM (*p* < 0.05; Figure 2C,D) of SNI rats. EA treatment led to a significant reduction in extracellular discharge frequency in these two brain regions (*p* < 0.05; Figure 2A–D).

The neurons activation of NTS (Figure 2E) and RVLM (Figure 2F) was also assessed by using immunofluorescence, which yielded the same results (Figure 2G~J). EA significantly reduced the expression of c‐Fos in the NTS (*p* < 0.05; Figure 2G,I) and RVLM (*p* < 0.05; Figure 2H,J). The above results showed that the activity of NTS and RVLM was the same as sympathetic nerve in EA analgesia, suggesting that NTS and RVLM played an important role in sympathetic regulation and EA analgesia.

### 3.3 Glutamatergic Neurons in the NTS and Presympathetic Neurons in the RVLM Were Activated

**FIGURE 3:**
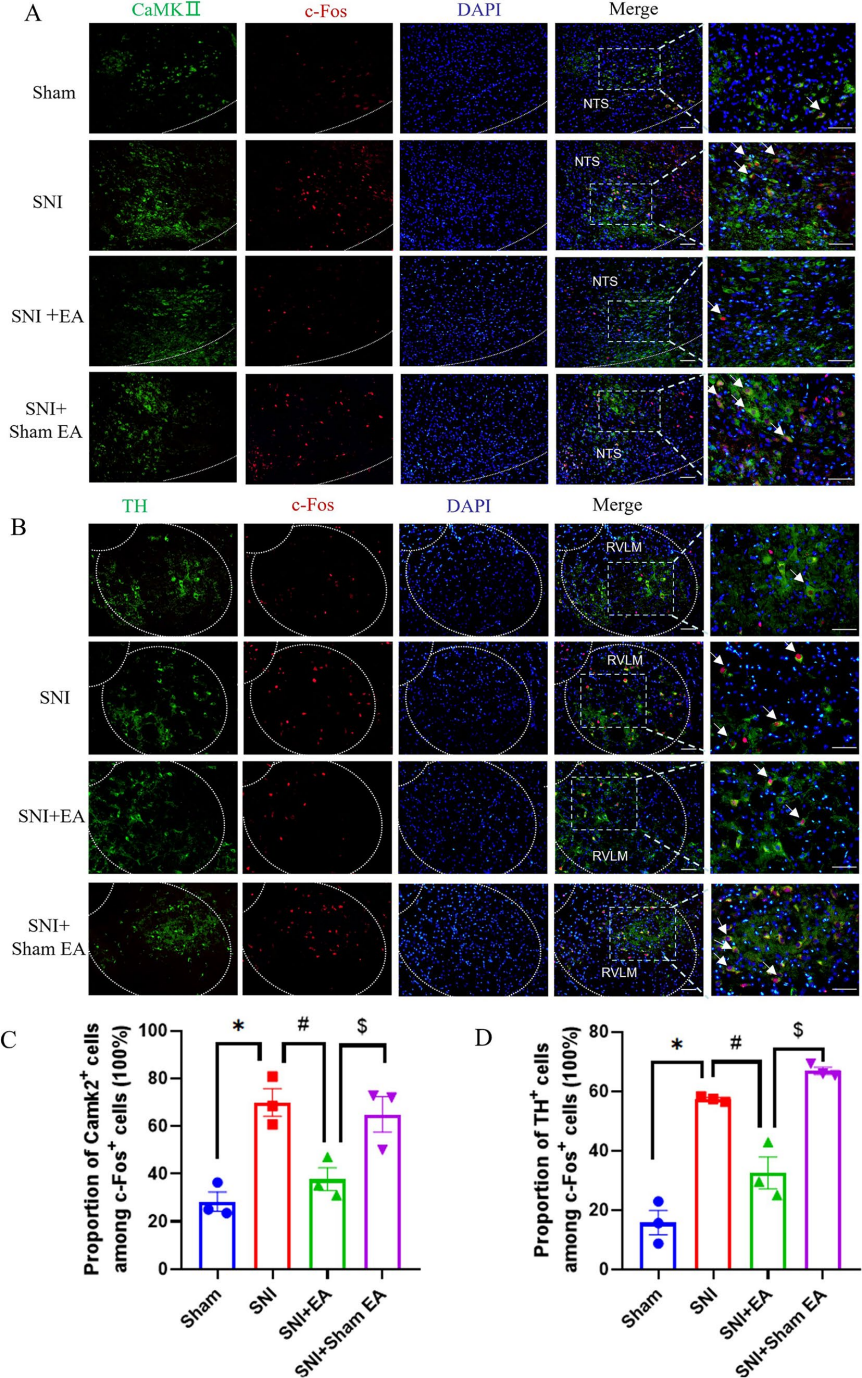
Validated the types of neurons in NTS and RVLM. Representative images of CaMKIIα (green) co‐stained with c‐Fos (red) in the NTS (A) and TH (green) costained with c‐Fos (red) in the RVLM (B) from the sham, SNI, SNI + EA, and SNI + sham EA groups; scale bar, 50 μm, the arrows indicated CaMKII^+^c‐Fos^+^ neurons in A and TH^+^c‐Fos^+^neurons in B. Proportion of neurons expressing CaMKIIα among c‐Fos in the NTS (C) and proportion of neurons expressing TH among c‐Fos in the RVLM (D) (n=3/group), one‐way ANOVA with Tukey in C and D. *p < 0.05 compared with sham rats, #*p* < 0.05 compared with SNI group, $*p* < 0.05 compared with SNI+sham EA group.

Neurons in the NTS and RVLM could classify into different types. After phenylephrine‐induced hypertension, 72% of Fos‐ir neurons were glutamatergic and 26% were GABAergic in rat NTS [44]. To identify the type of activated neurons in NTS and RVLM, immunofluorescence staining was selected. We found that in NTS, the c‐Fos^+^ neurons mainly coexpressed the glutamatergic neurons (Figure 3A,C), and in RVLM, the c‐Fos^+^ neurons mainly coexpressed presympathetic neurons (Figure 3B,D). The results further suggested that glutamatergic neurons in NTS and presympathetic neurons in RVLM might be involved in the regulation of sympathetic activity, as well as previous reports.

### 3.4 The NTS^Glu^ ‐RVLM Circuit Underlies the Effect of EA on Sympathetic Nerve Activity and Spontaneous Pain

**FIGURE 4:**
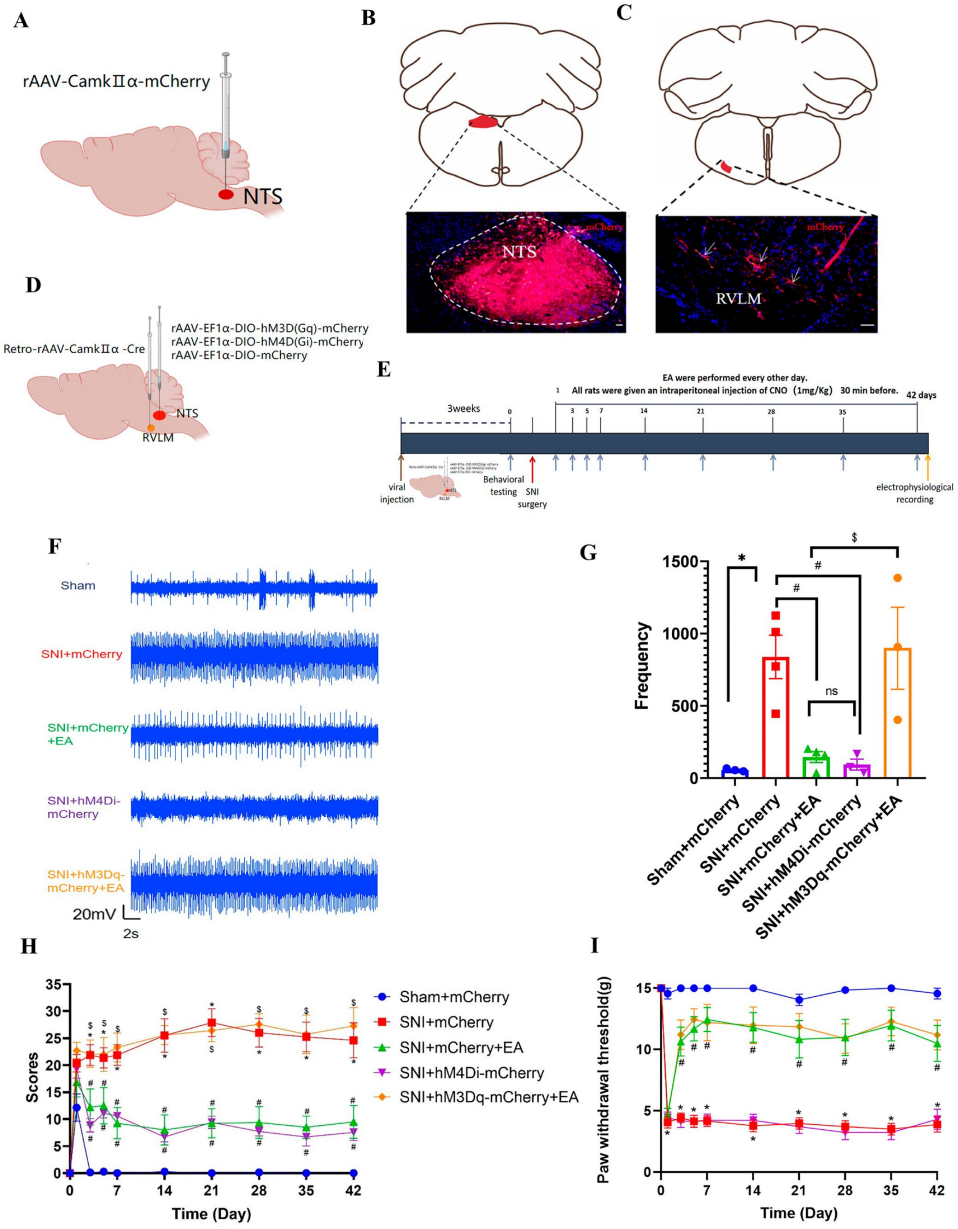
EA relieves spontaneous pain, not mechanical pain, through the NTS^Glu^‐RVLM circuit. (A) Schematic of the viral injection for anterograde tracing. (B) Representative photomicrograph of viral expression in the NTS. (C) mCherry signals in the RVLM; scale bar, 50 μm, the arrows indicated mCherry^+^ nerve fibers. (D) Schematic diagram of chemogenetics. (E) Schematic diagram of the experimental process. (F) Representative images of extracellular electrophysiology of RVLM from the sham + mCherry, SNI + mCherry, SNI + mCherry + EA, SNI + hM4Di‐mCherry, and SNI + hM3Dq‐mCherry + EA groups. (G) The discharge frequency of RVLM was significantly decreased after EA and chemogenetic inhibition of the NTS^Glu^‐RVLM circuit, the opposite effect of activating the NTS^Glu^‐RVLM circuit (*n* = 3 – 4/group), one‐way ANOVA with Tukey–Kramer test. (H) Time course of the SNI‐induced changes in the spontaneous pain scores (*n* = 7 – 8/group), using the Kruskal–Wallis test. (I)Time course of the SNI‐induced changes in the PWTs (*n* = 7 – 8/group), using the Kruskal–Wallis test. *p < 0.05 compared with the sham + mCherry group; #*p* < 0.05 compared with the SNI + mCherry group; $*p* < 0.05 compared with the SNI + mCherry + EA group; ns, not significant.

To study the role of the NTS^Glu^‐RVLM circuit on the EA regulation, we first determined whether glutamatergic neurons in the NTS project to the RVLM by injecting anterograde tracer virus rAAV‐CaMKIIα‐mCherry into the NTS in healthy male SD rats (Figure 4A). Successful injection was confirmed by localized expression of mCherry after 3 weeks (Figure 4B). The results indicated that CaMKII‐positive nerve fibers were observed in the RVLM (Figure 4C). These findings suggest a direct projection of NTS glutamatergic neurons to the RVLM. Subsequently, a chemogenetics virus was injected into the NTS and RVLM to manipulate the NTS^Glu^‐RVLM circuit (Figure 4D). The schematic diagram of the experimental process is shown in Figure 4E. The results showed that chemogenetic inhibition of the NTS glutamatergic neurons produced the same effect as EA, both of which could reduce extracellular discharge frequency in the RVLM (Figure 4F,G) and spontaneous pain scores (Figure 4H). In contrast, chemogenetic activation of the NTS glutamatergic neurons negated the effect of EA (Figure 4F–H). However, inhibition of the NTS^Glu^‐RVLM circuit had no effect on mechanical pain, and activation of the NTS^Glu^‐RVLM circuit also could not negate the effect of EA on mechanical pain (Figure 4I). Based on these findings, it is speculated that EA exerts an inhibitory effect on the NTS^Glu^‐RVLM neural circuit to modulate spontaneous pain but not mechanical pain.

**FIGURE 5:**
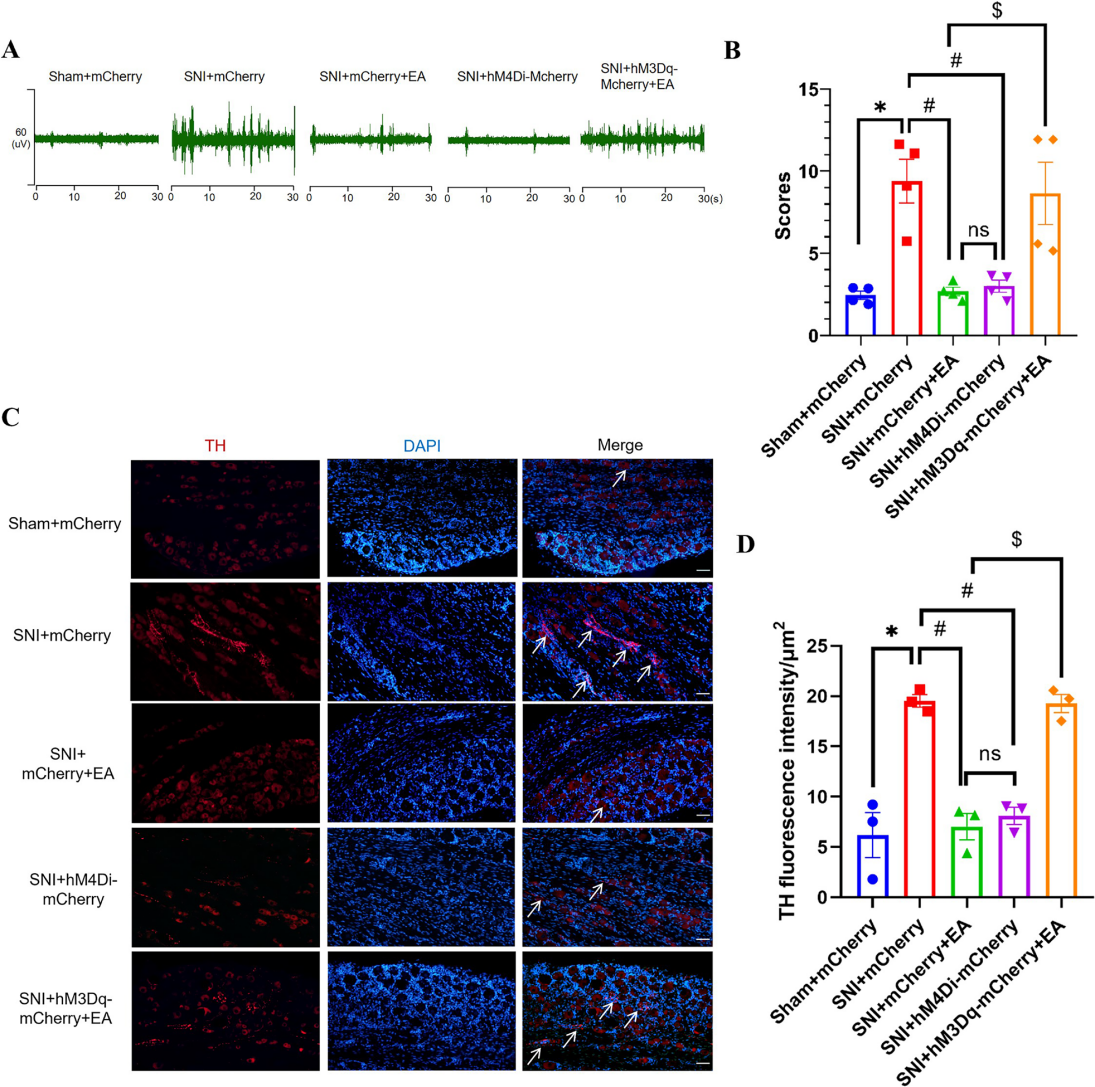
NTS^Glu^‐RVLM circuit underlies the regulation of EA on sympathetic. (A) Representative images of sympathetic nerve excitability from the sham + mCherry, SNI + mCherry, SNI + mCherry + EA, SNI + hM4Di‐mCherry, and SNI + hM3Dq‐mCherry + EA groups. (B) The sympathetic nerve excitability was significantly decreased after EA and chemogenetic inhibition of the NTS^Glu^‐RVLM circuit, opposite the effect of activating the NTS^Glu^‐RVLM circuit (*n* = 4/group), using the Kruskal–Wallis test. (C) Representative images of sympathetic sprouting in DRG from the sham + mCherry, SNI + mCherry, SNI + mCherry + EA, SNI + hM4Di‐mCherry, and SNI + hM3Dq‐mCherry + EA groups; scale bar, 50 μm, the arrows indicated TH^+^ nerve fibers. (D) The sprouting was significantly reduced after EA and chemogenetic inhibition of the NTS^Glu^‐RVLM circuit, opposite the effect of activating the NTS^Glu^‐RVLM circuit (*n* = 3/group), one‐way ANOVA with the Tukey test. *p < 0.05 compared with the sham + mCherry group; #*p* < 0.05 compared with the SNI + mCherry group; $*p* < 0.05 compared with the SNI + mCherry + EA group; ns, not significant.

In order to test whether the sympathetic nerve was involved in this discrepancy mediated by the NTS^Glu^‐RVLM circuit, sympathetic nerve activity and sprouting were analyzed under chemogenetic modulation and EA treatment. We found that chemogenetic inhibition of the NTS^Glu^‐RVLM circuit significantly inhibited the excitability (Figure 5A,B) and sprouting (Figure 5C,D) of sympathetic nerves, just like the effect of EA. Chemogenetic activation of the NTS^Glu^‐RVLM circuit could reverse the effect of EA on sympathetic nerves (Figure 5A–D). The results showed that the NTS^Glu^‐RVLM circuit contributed to the regulation of sympathetic nerves in EA treatment and might be the reason that NTS^Glu^‐RVLM circuit modulation only affected spontaneous but not mechanical pain.

## 4 Discussion

EA treatment has been widely used in alleviating neuropathic pain with fewer side effects. The previous study had validated that EA at GB30 and GB34 acupoints produces effects in relieving human and rodent pain behavior with neuropathic pain [45]. More than that, there were also studies that reported that 2 Hz EA treatment was effective in mice and rats SNI model [46, 47]. Besides, in order to avoid tolerance, we used 1‐2‐3 mA based on the reports from others [47, 48]. There was a study that reported that in order to establish a more stable comorbidity model of SNI neuropathic pain and depression, they gave the EA treatment every other day from 1 week post‐SNI until 6 weeks [49], although in SNI neuropathic pain, the EA treatment used in many labs was much shorter than that [46, 47]. In our pre‐experiment, we found that there was significant sympathetic nerve sprouting after 21 days of SNI surgery, but the obvious “basket‐like” structure had not yet emerged until 42 days after SNI and formed sympathetic‐sensory coupling (Figure S1). Combining literature and our own results, we picked GB30 and GB34 as the acupoints and used 2 Hz EA with 1–2‐3 mA in the study and selected 42 days post‐SNI surgery as the time point.

The contribution of the sympathetic nervous system to neuropathic pain has drawn more and more attention [50]. In the study, we found the excitability and sprouting of sympathetic nerves were increased in neuropathic pain; the results were consistent with previous researches [42]. EA treatment could not only relieve the pain behaviors but also inhibit the activity of sympathetic nerves, suggesting that sympathetic nerves mediated the EA analgesia [51]. However, in monoiodoacetate‐induced KOA rats, it was surprising that EA relieved synovitis and referred pain behaviors by increasing local sympathetic nerve and noradrenergic signaling in synovium [37]. The contradiction may be due to the sympathetic nerve in different locations playing different roles in pain conditions. Numerous studies have addressed the relationship between central NTS or RVLM and peripheral sympathetic nerves. In acute intermittent hypoxia (AIH), NTS is required for the maintenance of sympathetic long‐term facilitation [19]. Chemogenetic inhibition of NTS astrocytes completely abolished the potentiated sympathoexcitation induced by chronic intermittent hypoxia in rats [52]. Carotid body (CB) information that reaches the respiratory pattern generator could also raise sympathetic nerve activity by activating NTS glutamatergic neurons that target RVLM presympathetic neurons [53, 54]. The research demonstrated the activation of astrocytes and glutamatergic neurons in NTS mainly facilitated sympathetic activity, as well as there being a projection from NTS to RVLM. In our study, we further confirmed the projection from NTS glutamatergic neurons to RVLM by injecting anterograde tracer virus. Besides, we also found that in SNI rats, the increase of sympathetic activity and sprouting (Figure 1) was accompanied by the increase in the activity of NTS and RVLM (Figure 2).

Clinical neuropathic pain syndromes are characterized by evoked pain (mechanical pain) and spontaneous pain [55]. EA treatment alleviated both mechanical pain and spontaneous pain (Figure 1), while chemogenetic inhibition of NTS glutamatergic neurons to RVLM only alleviated spontaneous pain but not mechanical pain (Figure 4). Neurons in NTS and RVLM can be classified into different types [44]. In our research, neuronal activation was assessed by the presence of c‐Fos. We found that c‐Fos^+^CaMKII^+^ (glutamatergic neurons) labeled cells in NTS and their targeted c‐Fos^+^TH^+^ cells (presympathetic neurons) in RVLM were significantly increased in SNI rats (Figure 3), indicating that the NTS^Glu^‐RVLM circuit was activated in a pain condition. Glutamatergic neurons in NTS only involved depression‐like behaviors, but not hypersensitive behaviors in a chemotherapy‐induced neuropathic pain model [17]. In addition to the above‐mentioned, sympathetic neurons also underlie unique pain statuses. The development of neuropathic heat hyperalgesia required the sympathetic postganglionic neuron and alpha(2A) adrenoceptor, but the development of mechanical hyperalgesia did not require either sympathetic postganglionic neurons or the alpha(2A) adrenoceptor [56]. In Nav1.7‐dependent pain, distinct pain sensations require different sets of sensory and sympathetic neurons. Mechanical and inflammatory pain were abolished when ablating the Nav1.7 gene in all sensory neurons, but when the Nav1.7 gene was deleted only in nav1.8‐positive nociceptors, the heat‐evoked pain was retained [12]. In SNI mice, sympathetic activity and norepinephrine receptors were necessary for DRG cluster firing events, which correlated with spontaneous pain [43], although our results showed that sympathetic activity mediated by the NTS^Glu^‐RVLM circuit also contributed to spontaneous pain, there was a limitation that we did not observe their effects on cluster firing in DRG.

Central sensitization also contributed to EA analgesia in mechanical pain. In SNI mice, activation of CaMKII neuronal projections from the basolateral amygdala (BLA) to the rostral anterior cingulate cortex (rACC) and glutamatergic neuronal projections from the rACC to the dorsal raphe nucleus (DRN) mediated the analgesia of EA in mechanical pain [46, 57]. Besides, activation of glutamatergic neurons in the ventrolateral periaqueductal gray (vlPAG) also contributed to the analgesia of EA in SNI mechanical pain [58], but both chemogenetic activation of the rACC^Glu^‐vlPAG circuit and glutamatergic neuronal projections from the infralimbic (IL) to the BLA effectively blocked the analgesia of EA in SNI mechanical pain [59, 60]. Combined with our results, this further illustrates the concept that different neurons in different brain regions participate in distinct pain sensation during EA analgesia. Thus, chemogenetic inhibition and activation of NTS glutamatergic neurons only affect spontaneous pain and negate the effect of EA on it. On one hand, this might be due to the role of NTS glutamatergic neurons involved in different pain sensations; on the other hand, it might be due to chemogenetic inhibition and activation of NTS glutamatergic neurons affecting the neurons in the sympathetic system that only contribute to spontaneous pain, but more experiments are needed to confirm this concept in the future.

## 5 Conclusion

Our study found that sympathetic nerves in SNI model rats were abnormally excited. EA and chemogenetic inhibition of NTS glutamatergic neurons to RVLM inhibited sympathetic nerve activity and spontaneous pain, but not mechanical pain. Chemogenetic activation of NTS glutamatergic neurons to RVLM can negate the effect of EA. All results suggested that sympathetic nerve activity mediated by the NTS^Glu^‐RVLM circuit contributed to distinct pain sensations and was involved in EA analgesia.

## Author Contributions

W.C. and X.M. designed research, performed research, analyzed data, and wrote the paper with input from all authors. Y.‐M.F. gave help in analyzing the data, while C.‐Z.L., H.‐P.L., and G.‐X.S. performed research.

## Conflicts of Interest

The authors declare no conflicts of interest.

## Supporting information


**FIGURE S1** Sprouting of sympathetic nerve in SNI DRG. Representative images of sympathetic nerve sprouting. (A) The obvious sympathetic‐sensory coupling was observed in 6 weeks after SNI operation. (B) Sympathetic nerve mainly coupled with NF200 positive neurons, but not CGRP and IB4 positive neurons in SNI rats.

## Data Availability

The data that support the findings of this study are available on request from the corresponding author. The data are not publicly available due to privacy or ethical restrictions.
