# Supplementary figures and images for: Electroacupuncture Regulates Sympathetic Nerve Through the NTSGlu ‐RVLM Circuit to Relieve Spontaneous Pain in SNI Rats
Source: CNS Neurosci Ther. 2025 Mar 27;31(3):e70327. doi: 10.1111/cns.70327 (PMC11949842; doi:10.1111/cns.70327)

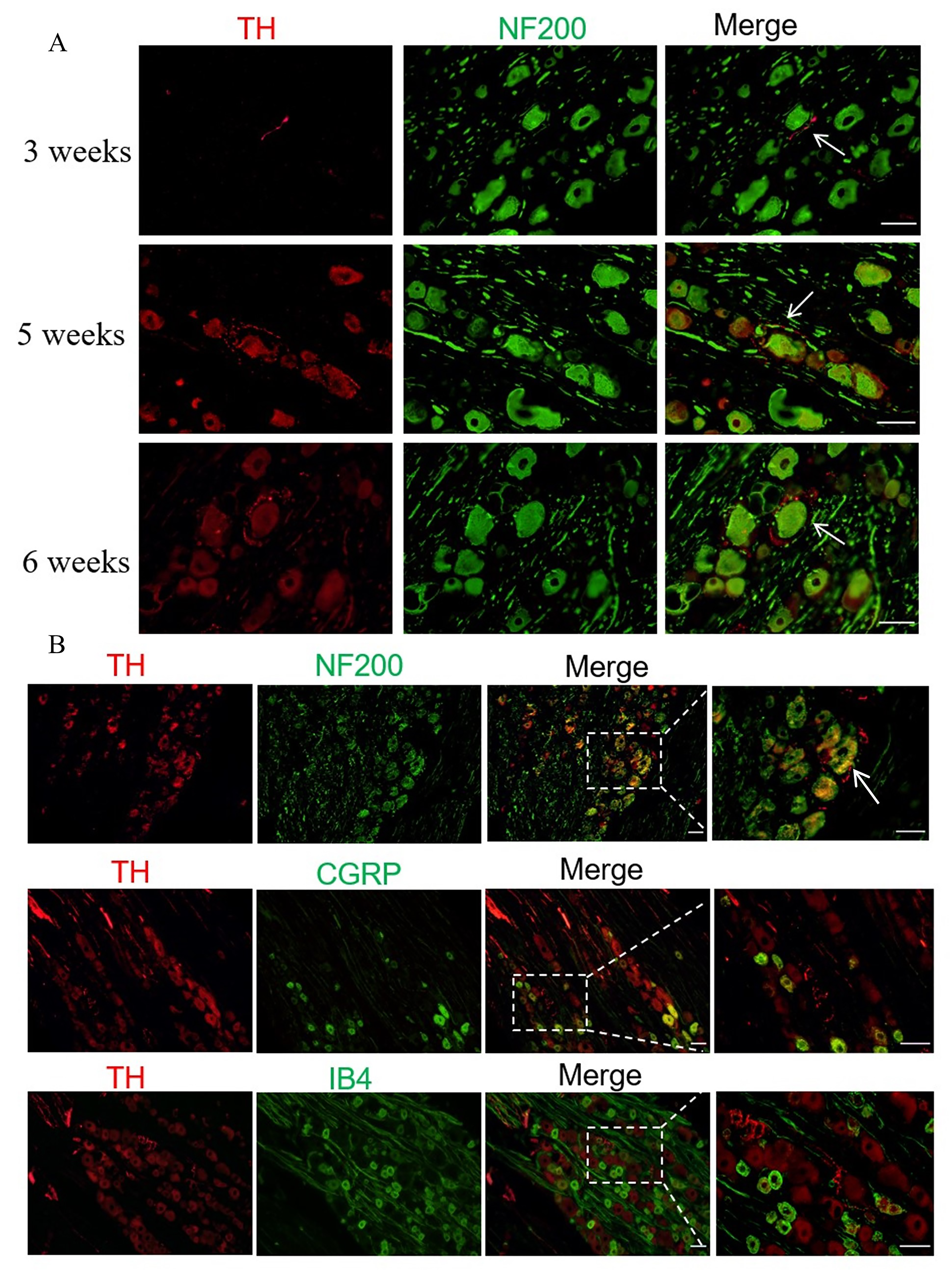

Supplement: Supplementary file 1 — FIGURE S1 Sprouting of sympathetic nerve in SNI DRG. Representative images of sympathetic nerve sprouting. (A) The obvious sympathetic‐sensory coupling was observed in 6 weeks after SNI operation. (B) Sympathetic nerve mainly coupled with NF200 positive neurons, but not CGRP and IB4 positive neurons in SNI rats. [file CNS-31-e70327-s001.tif]
